# The genomic architecture of resistance to *Campylobacter jejuni* intestinal colonisation in chickens

**DOI:** 10.1186/s12864-016-2612-7

**Published:** 2016-04-18

**Authors:** A. Psifidi, M. Fife, J. Howell, O. Matika, P. M. van Diemen, R. Kuo, J. Smith, P. M. Hocking, N. Salmon, M. A. Jones, D. A. Hume, G. Banos, M. P. Stevens, P. Kaiser

**Affiliations:** The Roslin Institute and Royal (Dick) School of Veterinary Studies, University of Edinburgh, Easter Bush, Midlothian, EH25 9RG UK; The Pirbright Institute, Genetics & Genomics Group, Surrey, GU240NF UK; Jenner Institute, Nuffield Department of Clinical Medicine, The Centre for Cellular and Molecular Physiology, Roosevelt Drive, Headington, Oxford, OX3 7BN UK; School of Veterinary Medicine and Science, University of Nottingham, Sutton Bonington Campus, Leicestershire, LE12 5RD UK; Scotland’s Rural College, Edinburgh, Easter Bush, Midlothian, EH25 9RG UK

**Keywords:** *Campylobacter*, Chicken, Resistance, Back-cross, Advanced intercross, Genome-wide association, Quantitative trait

## Abstract

**Background:**

*Campylobacter* is the leading cause of foodborne diarrhoeal illness in humans and is mostly acquired from consumption or handling of contaminated poultry meat. In the absence of effective licensed vaccines and inhibitors, selection for chickens with increased resistance to *Campylobacter* could potentially reduce its subsequent entry into the food chain. *Campylobacter* intestinal colonisation levels are influenced by the host genetics of the chicken. In the present study, two chicken populations were used to investigate the genetic architecture of avian resistance to colonisation: (i) a back-cross of two White Leghorn derived inbred lines [(6_1_ x N) x N] known to differ in resistance to *Campylobacter* colonisation and (ii) a 9^th^ generation advanced intercross (6_1_ x N) line.

**Results:**

The level of colonisation with *Campylobacter jejuni* following experimental infection was found to be a quantitative trait. A back-cross experiment using 1,243 fully informative single nucleotide polymorphism (SNP) markers revealed quantitative trait loci (QTL) on chromosomes 7, 11 and 14. In the advanced intercross line study, the location of the QTL on chromosome 14 was confirmed and refined and two new QTLs were identified located on chromosomes 4 and 16. Pathway and re-sequencing data analysis of the genes located in the QTL candidate regions identified potential pathways, networks and candidate resistance genes. Finally, gene expression analyses were performed for some of the candidate resistance genes to support the results.

**Conclusion:**

*Campylobacter* resistance in chickens is a complex trait, possibly involving the Major Histocompatibility Complex, innate and adaptive immune responses, cadherins and other factors. Two of the QTLs for *Campylobacter* resistance are co-located with *Salmonella* resistance loci, indicating that it may be possible to breed simultaneously for enhanced resistance to both zoonoses.

**Electronic supplementary material:**

The online version of this article (doi:10.1186/s12864-016-2612-7) contains supplementary material, which is available to authorized users.

## Background

*Campylobacter* is the leading cause of foodborne acute enteritis in humans in the developed world. The condition is usually self-limiting and symptoms last for 5–7 days, but in some cases the infection may be complicated by severe sequelae [1–3]. Epidemiology unequivocally implicates poultry as a key reservoir of human infection and up to 80 % of human cases may be attributable to the avian reservoir as a whole [4]. A recent year-long survey found *Campylobacter* in 73 % of chicken on retail sale in the United Kingdom [5]. There were 66,575 laboratory-confirmed human infections (mostly due to *Campylobacter jejuni*) and an estimated total of 685,000 cases in the UK in 2013 [6]. The number of *Campylobacter jejuni* (*C. jejuni*) in the caeca of chickens can exceed 10^8^ colony-forming units (CFU)/g and escape of gut contents and cross-contamination at slaughter is difficult to avoid. Quantitative risk assessments predict that even a relatively modest 2 log_10_ reduction in the number of *Campylobacter* in broiler carcasses could reduce the incidence of human disease due to infected chicken by up to 30-fold [7]. Therefore, a pressing need exists for strategies to reduce the entry of *Campylobacter* into the food chain. In the absence of effective licensed vaccines and inhibitors, selection for chickens with increased resistance to *Campylobacter* intestinal colonisation provides a sustainable complimentary control strategy.

There is a widely–held perception that *Campylobacter* is an inert commensal of birds*.* However, experimental infection of chickens with *Campylobacter* induces a rapid influx of heterophils (the avian functional equivalent of the mammalian neutrophil) into the gut and the production of pro-inflammatory cytokines and chemokines in the intestinal epithelium [8]. Maternal *C. jejuni*-specific antibodies protect chicks against experimental infection [9, 10] and are associated with the delayed incursion of *Campylobacter* into flocks [10, 11]. In some breeds of chicken, *C. jejuni* elicits prolonged inflammatory responses, damage to the intestinal mucosa, diarrhoea and failure to thrive [12, 13]. Conversely, both innate and acquired immune responses have been associated with differential resistance to *Campylobacter* intestinal colonisation [14–16].

The innate immune response to pathogen challenge and disease resistance varies between birds in inbred lines and outbred populations [12, 17–21]. The ability of *C. jejuni* to colonise the intestines differs amongst White Leghorn chicken inbred lines, with lines 6_1_ and N being at the extremes of phenotype [22]. An initial reciprocal backcross experiment between inbred lines 6_1_ and N revealed that the difference in bacterial numbers was heritable [22], but the host genetic mechanism of resistance to *Campylobacter* colonisation is not known. One published genome-wide association study (GWAS) of *C. jejuni* intestinal colonisation status (phenotypes analysed as a binary trait) in a novel dual-purpose chicken breed revealed one candidate locus on chromosome 11 near the *CDH13* gene [23]. There are also several studies of caecal gene expression analysis in chicken lines with different susceptibility to *Campylobacter* colonisation showing variation in transcription of genes influencing immune response [14, 15, 24].

The aim of the present study was to extend a previous investigation of inbred lines of chickens to determine the genetic architecture of resistance to *C. jejuni* colonisation using a focussed genotyping platform. This initially involved challenge of a back-cross population (*n* = 288) of White Leghorn chicken inbred lines 6_1_ and N with *C. jejuni* and genotyping of the birds for 1,243 fully informative single nucleotide polymorphism (SNP) markers. An independent replication study was then performed by challenging a 9^th^ generation advanced intercross line (AIL) population (*n* = 218) from a cross of the same two inbred lines with the same *C. jejuni* strain and genotyping with a 580 K SNP high density whole genome DNA array (Affymetrix^®^ Axiom^®^ HD) [25] to refine and identify new quantitative trait loci (QTLs). SNP markers significantly associated with *Campylobacter* intestinal colonisation resistance were detected on chromosomes 4, 7, 11, 14 and 16. We also performed pathway analysis and examined gene expression and re-sequencing data to identify candidate genes within the relevant genomic intervals.

## Results

### Phenotypes for parental lines, back-cross and AIL birds

Mean values and standard deviations of log-transformed caecal *C. jejuni* levels following experimental inoculation with strain 11168H for line 6_1_ and N parental birds, the [(6_1_ x N) x N] back-cross and the 9^th^ generation AIL (6_1_ x N) birds are listed in Additional file 1: Table S1. With a single exception of low counts on third day post-infection (dpi), no *C. jejuni* colonies were detected by direct plating of homogenates in any bird of resistant line 6_1._ In contrast, significant levels of *C. jejuni* colonisation were identified in susceptible line N birds, with the number of birds showing colonisation rising over time after infection. The results are consistent with the original report [22]. Resistance was semi-dominant in that levels of *C. jejuni* in the backcross and AIL population, measured five dpi, were intermediate between the levels seen in the two parental lines.

### Interval mapping and GWAS analysis of the back-cross experiment

**Fig. 1:**
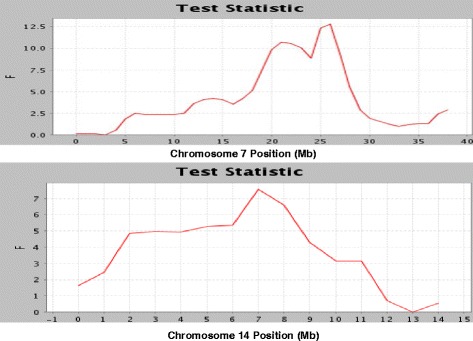
F-statistic scores obtained from least-squares interval mapping analysis in the back-cross experiment. F-statistic score of log-transformed number of *C. jejuni* per gram of caecal contents is plotted against location for chromosome 7 (above) and chromosome 14 (below)

The back-cross genotypes were analysed both using interval mapping (linkage analysis) and GWAS analysis (linkage disequilibrium (LD) analysis). In contrast to earlier studies [22, 23] we found with both analyses that levels of bacteria measured in challenged birds behaved as a quantitative trait, and mapped genetically to multiple loci. Two QTLs were detected on chromosomes 7 and 14 that were significantly associated with the log-transformed number of *C. jejuni* in the caeca at 5 dpi by the interval mapping analysis. The QTL on chromosome 7 was located at 26 Mb (Fig. 1) with a 1-LOD interval of 19.3 to 27.12 Mb. This QTL was significant at the chromosome-wide level (*P*-value <0.01) and, with an F value of 12.79, was close to genome-wide significance (5 % F-statistic threshold = 14.73). The QTL on chromosome 14 at 7 Mb (1 LOD interval 2.46 to 13.25 Mb) was significant at the chromosome-wide level (*P*-value <0.05) with an F value of 7.59 (Fig. 1).

GWAS analysis, using the limited informative marker set, identified both significant associations on chromosomes 7 and 14 as in the interval mapping analysis (Table 1). In addition, one SNP on chromosome 11 was also significantly associated at chromosome-wide level with the log-transformed caecal *C. jejuni* load (Table 1). The significant SNPs located on chromosome 7 were in high LD with each other (*r*^2^ = 0.89–0.98) and the same was the case for the SNPs on chromosome 14 (*r*^2^ = 0.97). Additional file 2: Figure S1 shows the Manhattan plot and the Q-Q plot displaying the GWAS results.

**Table 1:** List of SNPs associated with log-transformed caecal *Campylobacter* load at 5dpi in the back-cross population

SNP name	Chr	Position (bp)	*P*-value	-log_10_(P)
**Gga_rs15865889**	7	25741058	4.2 × 10^−4^	3.38
**Gga_rs14618024**	7	26003071	4.9 × 10^−4^	3.31
**Gga_rs16597361**	7	24812369	6.8 × 10^−4^	3.17
Gga_rs15010208	14	8288336	1.3 × 10^−3^	2.86
Gga_rs14076550	14	8716372	2.1 × 10^−3^	2.68
Gga_snp-142-64-19874-S-1	11	11791311	4.2 × 10^−3^	2.37

SNPs highlighted bold were significant at suggestive genome-wide level (*P* < 8.24 x 10^−4^) after Bonferroni correction

The SNPs in all three regions were confirmed to have a significant effect in the mixed model analysis (*P*-value <0.05). The additive genetic effects of the SNPs located on chromosome 7, 14 and 11 were log_10_ cfu/g 0.72 (*P*-value = 0.007), 0.71 (*P*-value = 0.006) and 0.68 (*P*-value = 0.009), respectively, and the phenotypic variance explained by these SNPs was 4.5 %, 4.3 % and 4.0 %, respectively.

### GWAS analyses of the AIL experiment

Multidimensional scaling analysis (MSA) revealed five substructure clusters in the AIL population, which were subsequently included in the GWAS model to correct results for population stratification.

**Fig. 2:**
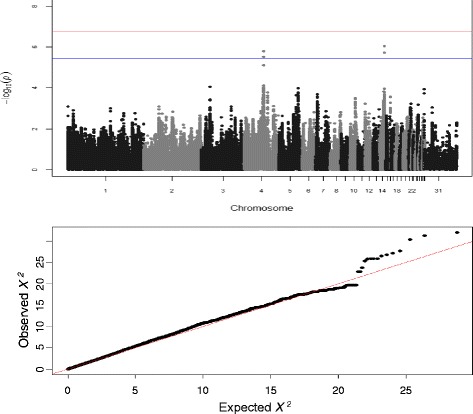
Manhattan plot and Q-Q plot displaying the GWAS results from the AIL experiment (continuous phenotypes). Genomic location is plotted against -log_10_(P) in the Manhattan plot (above). Genome-wide (*P* < 0.05) and suggestive genome-wide thresholds are shown as dashed lines. Q–Q plot (below) of observed *P*-values against the expected *P*-values for *Campylobacter* gut colonisation (log-transformed number of *C. jejuni* per gram of caecal contents)

GWAS analysis identified two SNPs significantly associated with the log-transformed number of *C. jejuni* in the caeca at 5 dpi on chromosome 14, located within the 1 LOD interval of the chromosome 14 QTL identified in the back-cross experiment (Table 2). Thus, the QTL on chromosome 14 was confirmed. Additionally, two SNPs crossing the suggestive genome-wide significant threshold were identified on chromosomes 4 and one SNP reaching the chromosome-wide significant threshold on chromosome 16 (Table 2). The Manhattan plot and the Q-Q plot for the GWAS results are displayed in Fig. 2.

**Table 2:** List of SNPs associated with caecal *Campylobacter* colonisation level at 5dpi in the AIL population

Phenotype	SNP name	Chr	Position (bp)	*P*-value	-log_10_P
Continues	**Affx-50646913**	14	12330355	9.09 × 10^−7^	6.05
	**Affx-50646912**	14	12329892	1.87 × 10^−6^	5.73
	**Affx-51436990**	4	50482802	1.61 × 10^−6^	5.80
	**Affx-51437092**	4	50540614	3.07 × 10^−6^	5.51
	Affx-50712088	16	216322	2.23 × 10^−4^	3.65
Binary	**Affx-51436990**	4	50482802	1.31 × 10^−7^	6.88
	**Affx-51437092**	4	50540614	5.57 × 10^−7^	6.25
	**Affx-51437128**	4	50570363	6.14 × 10^−7^	6.21
	**Affx-51437052**	4	50519407	1.46 × 10-^6^	5.83
	**Affx-51436951**	4	50458809	2.20 × 10^−6^	5.65
	**Affx-51436911**	4	50436442	2.20 × 10^−6^	5.65
	**Affx-51437087**	4	50538487	2.40 × 10^−6^	5.62
	**Affx-51437031**	4	50505712	3.24 × 10^−6^	5.49
	Affx-50711743	16	159629	2.15 × 10^−4^	3.66

Continues: log-transformed *Campylobacter* load in caeca; Binary: (0/1); SNPs in bold: significant at genome-wide (*P* ≤ 1.75 × 10^−7^) or suggestive genome-wide (*P* ≤ 3.50 × 10^−6^) level after Bonferroni correction

The estimated additive effects for SNP markers on chromosomes 14, 4 and 16 were log_10_cfu/g 1.2 (*P*-value = 0.01), 1.1 (*P*-value = 0.04) and 2.6 (*P*-value = 0.0001), respectively; proportions of the total phenotypic variance explained were 10, 9 and 6 %, respectively. Collectively, these three loci explained 25 % of the phenotypic variance.

The GWAS data was also reanalysed as a binary trait. This approach identified both the chromosome 4 and 16 significant associations with *Campylobacter* colonisation status (Table 2) and the significant SNP on chromosome 4 crossed the genome-wide significance threshold (Table 2). The Manhattan plot and the Q-Q plot for the GWAS results from the case-control analysis are displayed in Additional file 3: Figure S2.

### Annotation of QTL regions identified from the back-cross and AIL experiments

The large region encompassed by the QTL on chromosome 7 contains a relatively small number of genes, a total of 124 genes and 12 microRNA, inside the 1 LOD interval region (Additional file 4: Table S2).

The significant SNP on chromosome 11 is located in an intergenic region between two cadherin genes, cadherin 11 precursor (*CDH11*) and cadherin 8 (*CDH8*). Very close to this SNP a third cadherin gene, cadherin 5 (*CDH5*) was located (Additional file 4: Table S2).

The two significant SNPs identified on chromosome 14 in the AIL experiment were in strong LD and belonged to the same small LD block (154 bp) (Additional file 5: Figure S3). The two significant SNPs were in high LD with the other SNP markers located in regions 0.2 Mb upstream and downstream (Additional file 5: Figure S3). In this 0.4 Mb region, 22 genes and two microRNAs are located (Additional file 4: Table S2). The two significant markers were located in the intronic region of an undescribed gene in the chicken genome. Further investigation in the Ensembl database suggested that this was the orthologue of the N-acetyltransferase 15 (*NAT15*) gene in humans.

The significant SNPs on chromosome 4 were in high LD and located in a very small LD block (Additional file 6: Figure S4). These SNPs were also in high LD with SNP markers located in regions 0.2 Mb upstream and downstream (Additional file 6: Figure S4). In this 0.4 Mb region, five annotated genes and two microRNAs are located (Additional file 4: Table S2); all significant SNPs were located in the intronic region of the Ephrin receptor A5 (*EPHA5*) gene.

The significant SNP on chromosome 16 was located inside a single LD block with a length of 224 Kb and was in high LD with the other SNPs located there (Additional file 7: Figure S5). This region contains 29 annotated genes most of which are related in the Major Histocompatibility Complex (MHC) (Additional file 4: Table S2).

### Re-sequencing data analysis of back-cross and AIL results

To identify possible protein-coding genes associated with the detected QTLs, the genomic sequences of lines 6_1_ and N birds in the regions of interest were compared. The focus was on the identification of exonic single nucleotide variants (SNVs) with high importance (i.e., nonsense (stop-gain) and missense (non-synonymous) exonic and splicing), since these can affect the function of the gene leading to different isoforms of the transcribed proteins. Genomic regions located within 1 kb upstream of the respective genes were also analysed to identify SNVs with a potential regulatory effect. Due to the lack of regulatory element annotation in chickens, we developed our own pipeline for identifying possible regulatory site mutations. The 1 kb upstream genomic regions were scanned for putative TATA box and CpG island motifs. TATA boxes help facilitate transcription factor binding [26] thus TATA box mutations can effect transcription rates. CpG islands play an important role in methylation regulatory pathways [27]. CpG islands are characterised by regions which have a high density of CpG sites that can be methylated to down-regulate gene expression. Thus mutations in CpG sites can alter transcription regulation.

Summary statistics of all the SNVs identified in the candidate regions for *Campylobacter* colonisation resistance and all the SNVs detected, the Variant Effect Predictor annotation and the SIFT predictions are presented in Additional file 8: Figure S6 and Additional file 9: Table S3, respectively. In total, 20,125 variants were identified. The SNVs located in exonic regions were in total less than 5 % while the rest of the SNVs (95 %) were located in intronic, upstream and downstream regions. A few genes with SNVs that potentially could lead to non-functional transcripts were detected. More specifically, two genes *AXIN 1* located on chromosome 14 and *BG1* on chromosome 16 were found to contain a stop-gain SNV, a sequence variant whereby at least one base of a codon is changed, resulting in a premature stop codon, leading to a shortened transcript; three genes, *BG1* and *ENSGALG00000028367* located on chromosome 16 and *CCDC108* on chromosome 7 contained a splice acceptor variant, a splice variant that changes the 2 base region at the 3' end of an intron and might lead to splicing changes; two genes, *RACGAP1* and *SPEG* on chromosome 7 contained a splice donor variant, a splice variant that changes the 2 base region at the 5' end of an intron and can lead to splicing changes, as well. Furthermore, seventeen genes contained missense and according to SIFT prediction deleterious SNVs that might create partially or completely non-functional proteins. More specifically, *C16orf96* gene on chromosome 14, C4, *BFIV21, B-BTN2, TAP2* and *IL4I1* on chromosome 16*, IFIH1, LY75, SLC11A1, SLC38A11, SPEG, ZNF142, CCDC108, TTC21B, OBSL1, PTPRN, GLB1L* on chromosome 7 had missense deleterious SNVs. Several other genes contained SNVs with moderate impact. Details of genes containing splicing, 5′ UTR, both missense and UTR SNVs are presented in Additional file 10: Table S4.

TATA box motifs were identified in the upstream region of some genes but no variation was detected there. On the other hand, CpG island motifs were detected in many of the genes studied and some had SNVs. However, only in few cases the SNVs occur in CpG sites. Details of the genes containing SNVs in CpG sites are presented in the Additional file 10: Table S4.

The gene transcript from each experiment with the highest rate of non-synonymous coding SNVs (i.e., number of non-synonymous SNVs divided by the length of the coding DNA sequence (CDS) of the transcript, dN/L), rate of non-synonymous to synonymous SNVs (dN/dS), rate of exonic SNVs (number of exonic SNVs divided by exonic length of the transcript), rate of intronic SNVs (number of intronic SNVs divided by intronic length of the transcript) are presented in Table 3. These rates were considered to pertain to transcripts that differed between the two parental lines and an indication of positive selection that might result in the creation of different alleles responsible for functional differences in immune responses affecting disease resistance in the two lines.

**Table 3:** Genes and transcripts located in the QTL candidate regions for *Campylobacter* resistance with the highest variation among the two parental lines. (A) Backcross experiment (B) Advanced Intercross Experiment

Gene	Transcript	Chr	dN/dS	dN/L	exon_rate	intron_rate
A						
*ENSGALG00000026721*	ENSGALT00000043718	7	**3**	0.002441	0.003247	No introns
*FAM134A*	ENSGALT00000018495	7	**3**	0.002098	0.002797	0.006759
*RALB*	ENSGALT00000018997	7	**3**	0.004342	0.00519	0.00601
*IFIH1*	ENSGALT00000018067	7	**2**	0.000608	0.000743	0.000568
*ARPC2*	ENSGALT00000018675	7	**2**	0.001768	0.002418	0.003158
*PECR*	ENSGALT00000018735	7	**2**	0.002235	0.003007	0.006175
*LZTR1*	ENSGALT00000046043	7	**2**	0.002436	0.003925	0.003483
*NIFK*	ENSGALT00000019039	7	**2**	0.002424	0.002251	0.006897
*CCDC14*	ENSGALT00000019150	7	**2**	0.000729	0.001017	0.004697
*TTC21B*	ENSGALT00000038446	7	**1.75**	0.001702	0.002346	0.004126
*SMARCAL1*	ENSGALT00000018709	7	**1.333333**	0.001388	0.004471	0.005288
*C16orf96*	ENSGALT00000042897	14	**1.25**	0.008818	0.014139	0.00646
*COBLL1*	ENSGALT00000018021	7	**1.2**	0.001736	0.003179	0.003251
*OBSL1*	ENSGALT00000018337	7	**1.142857**	0.002852	0.00482	0.009001
*SLX4*	ENSGALT00000046089	14	**1**	0.001744	0.004729	0.005968
*SLC38A11*	ENSGALT00000018002	7	**1**	0.000749	0.003485	0.007136
*FIGN*	ENSGALT00000018023	7	**1**	0.000446	0.000892	No introns
*FAP*	ENSGALT00000018083	7	**1**	0.000444	0.001297	0.002828
*ASIC4*	ENSGALT00000018356	7	**1**	0.001254	0.002503	0.008669
*ZFAND2B*	ENSGALT00000044043	7	**1**	0.002137	0.004343	0.007585
*SLC11A1*	ENSGALT00000018510	7	**1**	0.00119	0.002381	0.009314
*C16orf96*	ENSGALT00000042897	14	1.25	**0.008818**	0.014139	0.00646
*ENSGALG00000023695*	ENSGALT00000039593	14	NA	**0.005587**	0.00534	0.002789
*RALB*	ENSGALT00000018997	7	3	**0.004342**	0.00519	0.00601
*GJD3*	ENSGALT00000018322	7	NA	**0.003421**	0.003421	0.008052
*OBSL1*	ENSGALT00000018337	7	1.142857	**0.002852**	0.00482	0.009001
*ENSGALG00000026721*	ENSGALT00000043718	7	3	**0.002441**	0.003247	No introns
*LZTR1*	ENSGALT00000046043	7	2	**0.002436**	0.003925	0.003483
*NIFK*	ENSGALT00000019039	7	2	**0.002424**	0.002251	0.006897
*ENSGALG00000023707*	ENSGALT00000039621	14	0.285714	**0.002304**	0.010333	0.008303
*CCDC108*	ENSGALT00000018523	7	0.75	**0.002268**	0.005093	0.009366
*PECR*	ENSGALT00000018735	7	2	**0.002235**	0.003007	0.006175
*TMEM169*	ENSGALT00000038136	7	1	**0.002176**	0.003236	0.007113
*ZFAND2B*	ENSGALT00000044043	7	1	**0.002137**	0.004343	0.007585
*FAM134A*	ENSGALT00000018495	7	3	**0.002098**	0.002797	0.006759
*CYP27A1*	ENSGALT00000003899	7	0.75	**0.001911**	0.004882	0.003762
*RACGAP1*	ENSGALT00000018359	7	0.6	**0.001808**	0.004455	0.007782
*NHEJ1*	ENSGALT00000018498	7	1	**0.001795**	0.011236	0.006046
*ARPC2*	ENSGALT00000018675	7	2	**0.001768**	0.002418	0.003158
*SLX4*	ENSGALT00000046089	14	1	**0.001744**	0.004729	0.005968
*COBLL1*	ENSGALT00000018021	7	1.2	**0.001736**	0.003179	0.003251
*C16orf96*	ENSGALT00000042897	14	1.25	0.008818	**0.014139**	0.00646
*MNR2*	ENSGALT00000018518	7	0	0	**0.012723**	0.00565
*NHEJ1*	ENSGALT00000018498	7	1	0.001795	**0.011236**	0.006046
*TUBA4A*	ENSGALT00000018477	7	0	0	**0.010549**	0.004069
*ENSGALG00000023707*	ENSGALT00000039621	14	0.285714	0.002304	**0.010333**	0.008303
*TUBA4A*	ENSGALT00000038318	7	0.058824	0.000729	**0.010204**	0.017134
*HSPBAP1*	ENSGALT00000019085	7	0.25	0.001374	**0.006512**	0.006158
*C2orf62*	ENSGALT00000029291	7	0.166667	0.000938	**0.006416**	0.008264
*ABCB6*	ENSGALT00000038272	7	0.2	0.000805	**0.006006**	0.007832
*ERCC3*	ENSGALT00000018775	7	0	0	**0.005964**	0.01652
*PLA2R1*	ENSGALT00000033111	7	0.5	0.00138	**0.005762**	0.008359
*STK16*	ENSGALT00000018480	7	0	0	**0.005722**	0.006693
*ENSGALG00000023695*	ENSGALT00000039593	14	NA	0.005587	**0.00534**	0.002789
*GLB1L*	ENSGALT00000018482	7	0.5	0.001586	**0.005227**	0.012599
*RALB*	ENSGALT00000018997	7	3	0.004342	**0.00519**	0.00601
*CCDC108*	ENSGALT00000018523	7	0.75	0.002268	**0.005093**	0.009366
*CYP27A1*	ENSGALT00000003899	7	0.75	0.001911	**0.004882**	0.003762
*EPB41L5*	ENSGALT00000018952	7	0	0	**0.004822**	0.006925
*OBSL1*	ENSGALT00000018337	7	1.142857	0.002852	**0.00482**	0.009001
*SPEG*	ENSGALT00000043042	7	0.194444	0.000763	**0.004805**	0.005843
*TUBA4A*	ENSGALT00000038318	7	0.058824	0.000729	0.010204	**0.017134**
*ERCC3*	ENSGALT00000018775	7	0	0	0.005964	**0.01652**
*GLB1L*	ENSGALT00000018482	7	0.5	0.001586	0.005227	**0.012599**
*RUFY4*	ENSGALT00000043062	7	0.333333	0.001133	0.003315	**0.01241**
*ATG9A*	ENSGALT00000019038	7	0	0	0.002891	**0.011987**
*RPL37A*	ENSGALT00000018702	7	0	0	0	**0.011268**
*C16orf5*	ENSGALT00000012346	14	0	0	0.002632	**0.011263**
*AAMP*	ENSGALT00000018663	7	0	0	0.001614	**0.010427**
*DES*	ENSGALT00000018446	7	0	0	0.003731	**0.010335**
*CTDSP1*	ENSGALT00000045561	7	0	0	0.001002	**0.010093**
*ANKZF1*	ENSGALT00000018493	7	0	0	0.004431	**0.010028**
*IGFBP2*	ENSGALT00000018698	7	0	0	0.003279	**0.009894**
*CCDC108*	ENSGALT00000018523	7	0.75	0.002268	0.005093	**0.009366**
*SLC23A3*	ENSGALT00000018510	7	1	0.00119	0.002381	**0.009314**
*OBSL1*	ENSGALT00000018337	7	1.142857	0.002852	0.00482	**0.009001**
*ASIC4*	ENSGALT00000018356	7	1	0.001254	0.002503	**0.008669**
*LY75*	ENSGALT00000018187	7	0.266667	0.000775	0.00358	**0.008563**
*PLA2R1*	ENSGALT00000033111	7	0.5	0.00138	0.005762	**0.008359**
*ENSGALG00000023707*	ENSGALT00000039621	14	0.285714	0.002304	0.010333	**0.008303**
*C2orf62*	ENSGALT00000029291	7	0.166667	0.000938	0.006416	**0.008264**
*ARHGEF1*	ENSGALT00000045992	7	0	0	0.002606	**0.008085**
B						
Gene	Transcript	Chr	dN/dS	dN/L	exon rate	intron rate
*BF2*	ENSGALT00000046170	16	**9**	0.00878	0.009381	0.010121
*BF2*	ENSGALT00000000139	16	**9**	0.00885	0.009452	0.01004
*BF2*	ENSGALT00000044107	16	**9**	0.00858	0.013504	0.010373
*BF2*	ENSGALT00000043207	16	**9**	0.008515	0.013432	0.01046
*RASSF6*	ENSGALT00000019068	4	**2**	0.001974	0.005961	0.006711
*BLB1*	ENSGALT00000008925	16	**2**	0.002994	0.002985	0
*TRIM27*	ENSGALT00000000172	16	**1.666667**	0.003915	0.006672	0.007874
*TAPBP*	ENSGALT00000000203	16	**1.333333**	0.00312	0.005109	0.010619
*C16orf96*	ENSGALT00000042897	14	**1.25**	0.008818	0.014139	0.00646
*TNXB*	ENSGALT00000000238	16	**1.25**	0.002016	0.003629	0.001132
*SLX4*	ENSGALT00000046089	14	**1**	0.001744	0.004729	0.005968
*TAPBP*	ENSGALT00000044889	16	**1**	0.002132	0.003899	0.010359
*SLX4*	ENSGALT00000012367	14	**0.875**	0.001439	0.004358	0.006141
*ENSGALG00000028367*	ENSGALT00000045620	16	**0.75**	0.002477	0.00578	0.013439
*TAP2*	ENSGALT00000000237	16	**0.666667**	0.00382	0.009537	0.014909
*BG1*	ENSGALT00000045385	16	**0.6**	0.005976	0.016882	0.010126
*TRAP1*	ENSGALT00000012459	14	**0.5**	0.000965	0.003201	0.007825
*HLADMB*	ENSGALT00000041213	16	**0.5**	0.002865	0.008137	0.006479
*TCLEC2D*	ENSGALT00000000183	16	**0.5**	0.001789	0.005938	0.011905
*TRIM41*	ENSGALT00000045935	16	**0.5**	0.001744	0.004208	0.00173
*TRIM27*	ENSGALT00000042650	16	**0.5**	0.000892	0.002676	0.003241
*BNK*	ENSGALT00000000188	16	NA	**0.013462**	0.011445	0.003878
*BF2*	ENSGALT00000000139	16	9	**0.00885**	0.009452	0.01004
*C16orf96*	ENSGALT00000042897	14	1.25	**0.008818**	0.014139	0.00646
*BF2*	ENSGALT00000046170	16	9	**0.00878**	0.009381	0.010121
*BF2*	ENSGALT00000044107	16	9	**0.00858**	0.013504	0.010373
*BF2*	ENSGALT00000043207	16	9	**0.008515**	0.013432	0.01046
*BG1*	ENSGALT00000045385	16	0.6	**0.005976**	0.016882	0.010126
*ENSGALG00000023695*	ENSGALT00000039593	14	NA	**0.005587**	0.00534	0.002789
*TRIM27*	ENSGALT00000000172	16	1.666667	**0.003915**	0.006672	0.007874
*TAP2*	ENSGALT00000000237	16	0.666667	**0.00382**	0.009537	0.014909
*ZNF692*	ENSGALT00000031515	16	0.454545	**0.003215**	0.00949	0.01307
*Hep21*	ENSGALT00000000164	16	NA	**0.003145**	0.00316	0.008
*TAPBP*	ENSGALT00000000203	16	1.333333	**0.00312**	0.005109	0.010619
*BLB1*	ENSGALT00000008925	16	NA	**0.002994**	0.002985	0
*HLADMB*	ENSGALT00000041213	16	0.5	**0.002865**	0.008137	0.006479
*DMB2*	ENSGALT00000000222	16	0.4	**0.002581**	0.010393	0.014409
*ENSGALG00000028367*	ENSGALT00000045620	16	0.75	**0.002477**	0.00578	0.013439
*ENSGALG00000023707*	ENSGALT00000039621	14	0.285714	**0.002304**	0.010333	0.008303
*TAPBP*	ENSGALT00000044889	16	1	**0.002132**	0.003899	0.010359
*TNXB*	ENSGALT00000000238	16	1.25	**0.002016**	0.003629	0.001132
*RASSF6*	ENSGALT00000019068	4	2	**0.001974**	0.005961	0.006711
*BG1*	ENSGALT00000045385	16	0.6	0.005976	**0.016882**	0.010126
*C16orf96*	ENSGALT00000042897	14	1.25	0.008818	**0.014139**	0.00646
*BF2*	ENSGALT00000044107	16	9	0.00858	**0.013504**	0.010373
*BF2*	ENSGALT00000043207	16	9	0.008515	**0.013432**	0.01046
*BNK*	ENSGALT00000000188	16	NA	0.013462	**0.011445**	0.003878
*DMB2*	ENSGALT00000000222	16	0.4	0.002581	**0.010393**	0.014409
*ENSGALG00000023707*	ENSGALT00000039621	14	0.285714	0.002304	**0.010333**	0.008303
*TAP2*	ENSGALT00000000237	16	0.666667	0.00382	**0.009537**	0.014909
*LOC422654*	ENSGALT00000041397	4	0	0	**0.009533**	0
*ZNF692*	ENSGALT00000031515	16	0.454545	0.003215	**0.00949**	0.01307
*BF2*	ENSGALT00000000139	16	9	0.00885	**0.009452**	0.01004
*BF2*	ENSGALT00000046170	16	9	0.00878	**0.009381**	0.010121
*HLADMB*	ENSGALT00000041213	16	0.5	0.002865	**0.008137**	0.006479
*DMB2*	ENSGALT00000041214	16	0	0	**0.008121**	0.011547
*TAP1*	ENSGALT00000045907	16	0.230769	0.001726	**0.006978**	0.010147
*TRIM27*	ENSGALT00000000172	16	1.666667	0.003915	**0.006672**	0.007874
*IL4I1*	ENSGALT00000000109	16	0.428571	0.001912	**0.006361**	0.005435
*RASSF6*	ENSGALT00000019068	4	2	0.001974	**0.005961**	0.006711
*TCLEC2D*	ENSGALT00000000183	16	0.5	0.001789	**0.005938**	0.011905
*ENSGALG00000028367*	ENSGALT00000045620	16	0.75	0.002477	**0.00578**	0.013439
*TAP2*	ENSGALT00000000237	16	0.666667	0.00382	0.009537	**0.014909**
*DMB2*	ENSGALT00000000222	16	0.4	0.002581	0.010393	**0.014409**
*ENSGALG00000028367*	ENSGALT00000045620	16	0.75	0.002477	0.00578	**0.013439**
*ZNF692*	ENSGALT00000031515	16	0.454545	0.003215	0.00949	**0.01307**
*TCLEC2D*	ENSGALT00000000183	16	0.5	0.001789	0.005938	**0.011905**
*DMB2*	ENSGALT00000041214	16	0	0	0.008121	**0.011547**
*CXCLi1*	ENSGALT00000019072	4	0	0	0.001606	**0.011457**
*C16orf5*	ENSGALT00000012346	14	0	0	0.002632	**0.011263**
*TAPBP*	ENSGALT00000000203	16	1.333333	0.00312	0.005109	**0.010619**
*BF2*	ENSGALT00000043207	16	9	0.008515	0.013432	**0.01046**
*BF2*	ENSGALT00000044107	16	9	0.00858	0.013504	**0.010373**
*TAPBP*	ENSGALT00000044889	16	1	0.002132	0.003899	**0.010359**
*TAP1*	ENSGALT00000045907	16	0.230769	0.001726	0.006978	**0.010147**
*BG1*	ENSGALT00000045385	16	0.6	0.005976	0.016882	**0.010126**
*BF2*	ENSGALT00000046170	16	9	0.00878	0.009381	**0.010121**
*BF2*	ENSGALT00000000139	16	9	0.00885	0.009452	**0.01004**
*BMA1*	ENSGALT00000000214	16	0.25	0.001274	0.005488	**0.010015**
*C4*	ENSGALT00000044830	16	0.421053	0.001616	0.005525	**0.008507**
*DNASE*	ENSGALT00000039621	14	0.285714	0.002304	0.010333	**0.008303**
*TRAP1*	ENSGALT00000012459	14	0.5	0.000965	0.003201	**0.007825**
*RASSF6*	ENSGALT00000019068	4	2	0.001974	0.005961	**0.006711**

Non synonymous SNVs/synonymous SNVs(dN/dS); non synonymous SNVs/CDS length (dN/L); NA = no synonymous SNV present; with bold is the rate based on which the genes were ranked

### Ingenuity pathway analysis of back-cross and AIL results

**Fig. 3:**
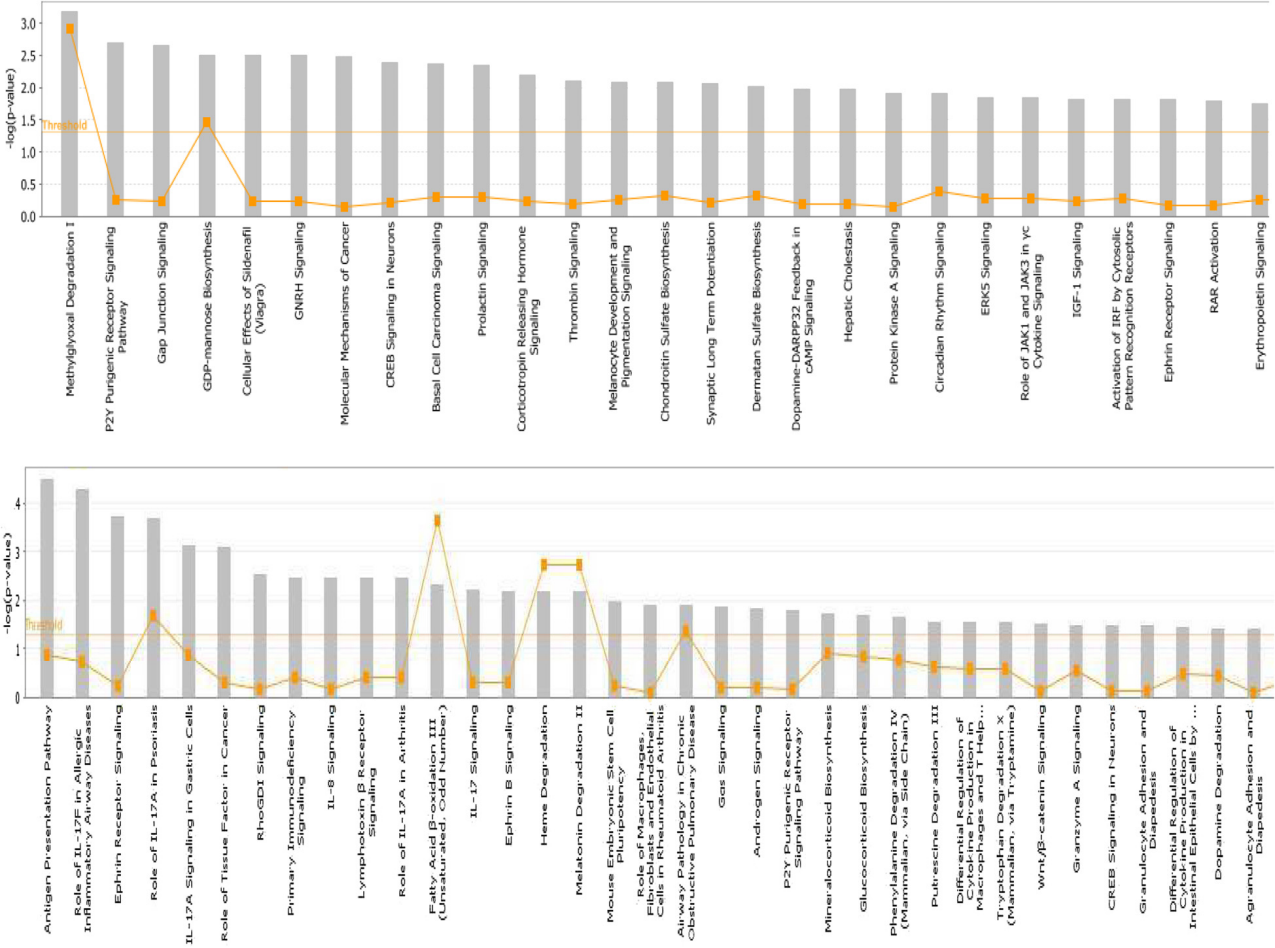
Pathway analysis using the IPA software. The most highly represented canonical pathways of genes located at the candidate regions for *Campylobacter* colonisation resistance derived from the back-cross (above) and the advance intercross line (below) experiments. The solid yellow line represents the significance threshold. The line with squares represents the ratio of the genes represented within each pathway to the total number of genes in the pathway

To identify potential canonical pathways and networks underlying the QTLs detected, we performed pathway analysis using the genes located in these regions. Pathways involved in innate and adaptive immune response, inflammatory response, response to infectious diseases, cell signalling and adhesion, and metabolism constituted the majority of the pathways highlighted for both back-cross and AIL results (Fig. 3). Moreover, two networks of molecular interactions related to immune response were constructed using the list of candidate genes for AIL (Additional file 11: Figure S7).

### Gene expression analysis

Many quantitative traits are associated with altered gene expression rather than coding variation. For example, variable expression of the satiety signal receptor, *CCKAR*, is associated with appetite control in chickens [28]. Two chemokine genes, *CXCLi1* and *CXCLi2*, lie in close proximity with the significant markers identified on chromosome 4. Both chemokines are induced after *Campylobacter* infection in chickens [8, 12, 29] and might be involved in effective host immune response. We therefore examined their expression in caecal tonsils of challenged birds. The data are shown in Additional file 12: Figure S8. Data are expressed as the fold change in mRNA levels when samples from infected birds were compared to non-infected birds of the same age from each line. In *C. jejuni* infected birds from both lines *CXCLi1* and *CXCLi2* were down-regulated. Nevertheless, the level of chemokine *CXCLi2* was decreased in line N significantly (*P*-value <0.05) more than in line 6_1_, while *CXCLi1* levels did not significantly differ between the lines.

### Selection of candidate genes

A total of 20 genes were selected amongst all genes located in the regions of interest identified from the analysis of the back-cross and AIL populations as good candidate genes for avian resistance to *Campylobacter* colonisation (Additional file 13: Table S5). Gene selection was based on their biological function, proximity to significant markers, sequencing differences, mRNA expression (tested for *CXCLi1* and *CXCLi2*) after *Campylobacter* infection, their involvement in immune response pathways and networks, and any previously known involvement in other infectious diseases in poultry (Additional file 9: Table S4).

## Discussion

The present study indicates that the precise level of *Campylobacter* intestinal colonisation is a heritable complex quantitative trait under the genetic control of multiple loci, genes and linked sequence variants. In the back-cross analysis we identified candidate QTLs in three genomic locations on chromosomes 7, 11 and 14. Using a 9^th^ generation AIL population we refined the location of the QTL identified on chromosome 14 (from a 12 Mb region to a 0.4 Mb region) and detected two additional QTLs located on chromosomes 4 and 16. The initial candidate regions identified in the back-cross experiment have limited resolution and the detected QTLs may reflect the effect of many linked variants which are separated when the LD blocks break after many generations of recombination [30]. The identification of new QTLs in the AIL analysis could be attributed to genotyping based on many more markers (approximately 300,000 compared to 1,300 in the back-cross experiment), which considerably increased the power of QTL detection.

Two good candidate genes, *TRAP 1* and *AXIN 1* were identified in the refined QTL location on chromosome 14; this exemplifies the common findings from the two experimental designs (back-cross and AIL). *TRAP1* is coding for a mitochondrial heat shock protein with antioxidant and anti-apoptotic functions [31, 32]. Our re-sequencing data revealed that *TRAP1* is divergent between the two lines. It has a high SNV/L and dN/dS rate that may affect its function and also has 3′/5′ UTR SNV that may impact on gene expression. *AXIN 1*, is coding for a scaffolding protein controlling the levels of β-catenin, which in turn regulates NF-kB activity. This gene is also very divergent in the two lines with a stop/coding SNV, a high dN/L and dN/dS rate as well as UTR SNV.

The two parental lines, 6_1_ and N, have a different MHC haplotype, B2 and B21, respectively. Therefore, the involvement of MHC, a genomic region that encodes molecules that provide the context in which T cells recognize foreign antigens, in *Campylobacter* colonisation resistance, is not unexpected. This genomic region is the focus of considerable interest because of the strong, reproducible infectious disease associations found with specific MHC haplotypes [33–36]. However, the highly polymorphic nature of the genes, the strong LD that exists within this region [37–39], and the limited number of SNP markers on chromosome 16 present obstacles to associating individual genes with disease responses. The previous study of the inbred lines suggested that MHC was not involved [22], but this was based on only 41 back-cross (between lines 6_1_ and N) birds and the genotyping was performed using only one microsatellite marker located close to the MHC. The study of Boyd et al. inferred autosomal dominance at a single locus, but that conclusion was based upon the failure to detect the differential colonisation between the parent line 6_1_ and crossed birds [22].

As noted in the introduction, the only previous GWAS study of *Campylobacter* intestinal colonisation resistance in chickens [23], identified a risk locus on chromosome 11 associated with the T-cadherin (*CDH13*) gene. This finding might be linked to the cadherin genes (*CDH5, CDH11, CDH8*) identified in our back-cross analysis. Cadherins are a super-family of calcium-dependent proteins, with a significant role in cell-cell adhesion and the maintenance of structural and functional tissue integrity [40]. The cadherins might interact with *C. jejuni* and consequentially affect resistance to colonisation either by facilitating internalisation or inciting a protective host response [41–44].

Connell et al. examined gene expression of caeca tissue from high colonised and nil colonised birds of the same breed challenged with *Campylobacter*, and found evidence of more rapid innate immune response to the infection in nil colonised birds [14]. Among the candidates genes identified in the current study, there are two genes associated with two major pro-inflammatory chemokines (*CXCLi1* and *CXCLi2*) that are reportedly induced during *Campylobacter* infection both in chickens and humans [45, 46]. Stimulated heterophils produce *CXCLi1* and *CXCLi2* to induce leukocyte (primarily heterophil) chemotaxis [47, 48]. Re-sequencing data analysis of the two parental inbred lines (6_1_ and N) revealed no protein-coding variation, but did identify an SNV in the 3′UTR of *CXCLi2*. Although SNV located in the regulatory regions of individual genes are of high importance, relevant knowledge in chickens is very limited and literature virtually non-existent. Whether this particular variant or more distal control elements are responsible, direct measurement of mRNA levels in caecal tonsils of infected birds confirmed differential expression of *CXCLi2* between the two lines. Similarly, Connell et al. detected a differential expression only for *CXCLi2* [14]. According to these results *CXCLi2* may plausibly contribute to resistance to *Campylobacter* colonisation in chickens.

A previous study of SNP associations with innate and adaptive immune responses in laying hens identified a QTL on chromosome 7 at the same region where the peak of our QTL on the same chromosome is located, associated with complement activity [49]. Several other innate immune genes, very different at sequence level between the two inbred lines, lie under the peak of our identified QTL within the 1 LOD-drop confidence interval. The interferon-induced helicase C domain-containing protein 1 (*IFIH1*) gene with a predicted splicing SNV is located at 20.5 Mb, the lymphocyte antigen 75 precursor (*LY75*) gene, also known as *CD205*, which encodes a receptor on dendritic cells, is located at 21.5 Mb. The natural resistance-associated macrophage protein 1 *(NRAMP1)* gene, also known as *SLC11A1*, located at 22 Mb, is another potentially exciting candidate gene to explain this QTL. *NRAMP1* regulates intracellular pathogen proliferation and macrophage inflammatory responses, by influencing the phagolysosomal function of macrophages. Variants in this gene have been associated with resistance to *Salmonella* infection in mice and poultry [50, 51]. Interestingly, host resistance to *C. jejuni* infection in the mouse is also *NRAMP1*-dependent [52].

Inbred lines 6_1_ and N are resistant and susceptible respectively not only to *Campylobacter* gut colonisation, but also to *Salmonella* gut colonisation, supporting speculation that the genetic control of colonisation could be at least partly common for these bacteria [53]. Extensive QTL mapping studies have taken place to identify loci for resistance to *S. enterica* serovar Typhimurium and Enteritidis colonisation over the last 20 years using these two inbred chicken lines [2, 20, 51, 54, 55]. Interestingly, two QTLs for resistance to enteric carriage of *Salmonella* have been identified at the same regions on chromosome 14 [54] and 16 [51, 55] as in our study. Both *TRAP1* and *AXIN 1*, the two putative candidate genes on chromosome 14 for *Campylobacter* colonisation resistance, have been also associated with *Salmonella* infection in previous studies [56, 57].

Line 6_1_ is also resistant to infectious bursal disease virus (IBDV) and Marek’s disease virus (MDV) while line N is resistant to infectious bronchitis virus (IBV) and MDV. Whole-genome gene expression analyses comparing resistant line 6_1_ with susceptible inbred line 7 for MDV and inbred Brown Leghorn [Brl] line for IBDV have been conducted to investigate the host response to these infections [58–63]. Likewise, similar analysis comparing resistant line N with susceptible inbred line 15I for IBV has been conducted. Among the genes highlighted as candidate genes for IBV, IBDV or MDV resistance, with different expression during the host response and differential expression between the resistant and susceptible lines, were many of the genes identified in our study as good candidates for *Campylobacter* intestinal colonisation resistance (Additional file 13: Table S5). These genes may play a central role in an overall effective host immune response.

## Conclusion

The multiple QTLs identified as well as the many immune response and inflammation pathways and the predicted involvement of MHC attest to a complex trait controlled by many candidate genes each with a moderate or weak effect. However, the magnitude of the additive effect size of the significant markers and the large proportion of the phenotypic variance explained by them is encouraging for informing breeding strategies for enhanced *Campylobacter* colonisation resistance in broiler chicken. A study by Gormley et al. revealed that levels of contamination generated by natural exposure varied by at least two orders of magnitude in a wide range of commercial broiler genotypes [64]. As our findings pertain to inbred lines and linkage disequilibrium will likely be different in outbred broiler populations future research is now warranted to explore the genetic basis for resistance in commercial birds and extent of variation at the QTLs identified. Our study highlights the utility of using inbred lines as a resource to map resistance-associated loci, gaining increased power to detect QTLs with modest effects, and informs the design of selective breeding strategies for control of a major zoonosis.

## Methods

### Ethical statement

All animal experiments were conducted in accordance with the Animals (Scientific Procedures) Act 1986, with the approval of the Ethical Review Committee of The Pirbright Institute (under project licence PPL 30/2462) and the Animal Welfare and Ethical Review Body of The Roslin Institute (under PPL 60/4420).

### Animals

Chicken inbred lines 6_1_ (resistant to *Campylobacter* colonisation) and N (susceptible to *Campylobacter* colonisation) were originally derived from White Leghorn flocks at the USDA-ARS Avian Disease and Oncology Laboratory in East Lansing, MI, USA. The lines have been maintained by random mating within the specified-pathogen-free (SPF) flocks at the Pirbright Institute in the UK since 1972 (line 6_1_) and 1982 (line N). To generate the back-cross (*n* = 288 for the present study), F1 progeny (6_1_ x N) were crossed with the susceptible line N.

Colonies of lines 6_1_ and N were given to INRA Tours by the Pirbright Institute over a decade ago. A 9^th^ generation AIL between lines 6_1_ and N was generated there. At each generation, animals from distinct families were crossed to minimise inbreeding and increase the number of recombinations as suggested by Darvasi and Soller [65]. In total, 218 AIL birds were generated for the present study.

### Bacterial challenge

In the parent lines, back-cross and AIL, day-old birds were orally inoculated with 0.1 ml of an overnight Mueller-Hinton broth culture of *Campylobacter*-free gut flora originally taken from the caecal contents of an adult SPF chicken. Birds were orally challenged at three weeks of age with 10^8^ CFU *C. jejuni* strain 11168H, a hypermotile variant of the sequenced strain NCTC11168 that readily colonises chickens [66] or with control Mueller-Hinton medium only. To confirm resistance or susceptibility to *Campylobacter* colonisation, control birds (*n* = 4–5 per line) and infected birds (*n* = 8–10 per line) were killed at 2, 3, 4 and 5 dpi and a sample of caecal contents was obtained. In addition, caecal tonsil samples of the birds killed 5 dpi were removed to RNAlater for RNA extraction, and gene expression analysis, as described below. Three separate challenge experiments were performed for logistical reasons on the back-cross and AIL populations to assess the level of C*ampylobacter* colonisation in which birds were killed at 5 dpi and caecal contents were removed to measure the *Campylobacter* load and blood samples were taken for DNA extraction and genotyping, as described below.

### Phenotyping

The level of bacterial colonisation in the caecal contents of infected birds was determined in all birds from the back-cross and AIL populations as well as the respective parents as described previously [67]. Bacterial counts for both the back-cross and AIL population were skewed and were accordingly log-transformed in order to normalise their distribution. Thus, phenotypes were expressed on a continuous scale as log-transformed bacterial counts. Moreover, in the case of AIL the phenotypes were also expressed on a binary (0/1) scale indicating absence or minimal colonisation (i.e., no colonies detected by direct plating of 0.1 g homogenate; thus corresponding to birds with *Campylobacte*r load ≤ 100 CFU/g of content)/presence of colonisation.

### Genotyping and QTL detection

This part of the study was conducted as two separate experiments described below.

### Back-cross experiment

#### QTL mapping analysis

Over 18,000 genome-wide SNPs were screened in the parent lines (6_1_ and N) to identify fully informative markers for the back-cross mapping study. All SNPs were available through Ensembl (www.ensembl.org) and were analysed using existing panels of chicken SNPs. Markers were selected in the parent lines on the basis of their fixation for the alternate allele at each position, providing maximum information content in the back-cross population. A total of 1,385 SNPs were selected from the 18,000 SNPs panel for analysis in the present study. Genotyping and quality control were performed as described before [20]. A full list of the SNPs (1,243) used in the final analysis is displayed in Additional file 14: Table S6. Positions of SNP markers were obtained using the Gal-gal4 assembly in Ensembl Genome Browser (www.ensembl.org).

QTL analysis was performed by regression interval mapping [68] using the QTL Express software [69] available through GRIDQTL (http://gridqt1.cap.ed.ac.uk) as described previously [20].

#### GWAS analysis

The back-cross genotypes were also analysed in a GWAS in order to compare with and validate interval mapping results. The following thresholds were used for quality control: minor allele frequency (MAF) < 0.05 and call rate > 95 %. Deviation from Hardy-Weinberg equilibrium was not considered as a criterion for excluding SNPs since this was a back-cross population. After the quality control, 1,212 SNP markers remained for further analysis. The software GEMMA [70] was used to run the GWAS analysis using a standard univariate linear mixed model in which sex (male, female) and experiment number (1, 2, 3) were fitted as fixed effects and the genomic relationship matrix among individuals was included as a random effect. A Bonferroni correction was applied for multiple testing [71]. After Bonferroni correction, significance thresholds were *P* ≤ 4.12 × 10^−5^ and *P* ≤ 8.24 × 10^−4^ for genome-wide (*P* ≤ 0.05) and suggestive (namely one false positive per genome scan) levels, corresponding to -log_10_(P) of 4.38 and 3.08, respectively. Searches for significant SNPs were performed also at chromosome-wide level (*P* ≤ 0.05).

#### SNP validation

Individual markers found to be significant in GWAS were further verified in an association analysis where each SNP was fitted as a fixed effect simultaneously with other fixed effects previously fitted (in the GWAS analysis) in a mixed model. The analysis was carried out with the software ASREML [72]. This analysis yielded estimates of the magnitude of the additive SNP effect, as the difference in value between the homozygous (line N-AA) and the heterozygous (AB) genotypes. Solutions were the predicted trait values for each genotype class.

Finally, to evaluate the extent of LD and identify potential regions of causal mutations for *Campylobacter* colonisation resistance, LD among SNPs was calculated as an r-square value using the software Plink [73]. Furthermore, LD blocks in the regions where significant SNPs were found with GWAS were visualised using the software Haploview [74].

### Advanced intercross line experiment

#### GWAS

AIL birds were genotyped using a 580 K SNP high density whole genome SNP array (Affymetrix^®^ Axiom^®^ HD) [74]. The SNP genotype data were subjected to quality control measures using the following thresholds: MAF < 0.02 and call rate < 95 %. Deviation from Hardy-Weinberg equilibrium was not considered as a method for excluding SNPs since this was an AIL population. After quality control, 286,432 SNP markers remained for further analysis. Positions of SNP markers were obtained using the Gal-gal4 assembly in Ensembl Genome Browser (www.ensembl.org).

The phenotypes were treated both as continuous and as binary data (based on a case-control model). In the case of the binary analysis, 133 birds with no or minimal colonisation where considered as controls and the remaining 85 birds were considered as cases.

The AIL data was analysed using GWAS. To investigate any population stratification present in the population, a genomic relationship matrix was generated from all individuals. This genomic relationship matrix was converted to a distance matrix that was used to carry out classical MSA. All analyses of population stratification were performed using GenABEL software [75]. The same software and the mixed model used in the back-cross was also used for the AIL analyses. In addition to sex and experiment, population cluster (1–5) was fitted as a fixed effect to account for population structure. A Bonferroni correction for multiple testing was applied. After Bonferroni correction significance thresholds were *P* ≤ 1.75 × 10^−7^ and *P* ≤ 3.50 × 10^−6^ for genome-wide (*P* ≤ 0.05) and suggestive levels, corresponding to -log_10_(P) of 6.75 and 5.45, respectively. In addition, a search for SNPs significant at the chromosome-wide level was performed.

#### SNP validation

Individual SNP markers that were significant at the genome- and chromosome-wide level in GWAS were further verified in an association analysis as described for the back-cross. This model also yielded estimates of the magnitude of the significant SNPs effects as described previously by [76].

Finally, the extent of LD among the significant SNP markers was calculated and LD blocks were built as described for the back-cross.

### Further analyses pertaining to both experiments

#### Annotation of the genes in the QTL candidate regions

The Biomart data mining tool within the Ensembl database (http://www.ensembl.org/biomart/martview/) and the Gal-gal4 assembly were used to locate genes in the candidate regions for *Campylobacter* colonisation resistance identified by the QTL mapping analysis and GWAS for the back-cross and AIL populations. In the case of GWAS, the limits of genomic candidate regions were determined by the LD block structure and the LD present among the significant SNP markers and the neighbouring markers.

The Variant Effect Predictor tool within the Ensembl database (http://www.ensembl.org/Tools/VEP) and the Gal-gal4 assembly were used to determine the location of the significant SNPs identified in the GWAS analyses on the reference genome. The effect of SNPs located in exonic regions on the protein sequence was determined with a SIFT prediction [77] of the protein sequence changes.

#### Re-sequencing data analysis

The two parental lines (6_1_ and N) were fully re-sequenced to 15–20 fold coverage, using pools of 10 individuals per line. Sequencing was performed on Illumina GAIIx platform using a paired end protocol [74]. Re-sequencing data of the candidate regions for *Campylobacter* colonisation resistance, derived from both the back-cross and AIL analysis results, was extracted. More specifically, the extracted sequence included the gene in question and 1 kb upstream and 1 kb downstream of the gene. The SNVs between the two parental lines and the reference genome in these regions were detected using the Mpileup tool for SNP calling (SAMtools v0.1.7). Detected SNVs were annotated using the VEP tool within the Ensembl database (http://www.ensembl.org/Tools/VEP). Information for all SNVs present in the regions of interest was collated. SNV that were in common in the two parental lines but different from the reference genome were filtered out. We separated SNVs into categories based on their relationship to genes. These categories were exonic, intronic, and 1000 bp upstream and downstream. Within the exonic category, there were additional subcategories of UTR and CDS. For SNV’s occurring in CDS regions we also predicted SNV effect as being synonymous, missense, nonsense (stop gain), or splicing. SIFT predictions of the effects of CDS SNVs (tolerated or deleterious) were also acquired through VEP. While mutations in transcribed regions can lead to altered transcriptional products, mutations in regulatory regions can affect transcription rates which ultimately alters phenotype. However, due to the lack of regulatory element annotation in chickens, we developed our own pipeline for predicting TATA boxes and CpG islands. Tata boxes are short DNA regions which act as Transcription Factor Binding sites. CGIs are interspersed DNA sequences that deviate significantly from the average genomic pattern by being GC-rich, and predominantly non-methylated. TATA boxes were predicted by looking for TATA box motifs identified in humans (Computational modeling of oligonucleotide positional densities for human promoter prediction) within the region 20–40 bp upstream of each gene. CpG islands were predicted by using 100 bp windows and scanning across the 1000 bp upstream region with 1 bp intervals to find >200 bp regions with >50 % GC content and an Observed/Expected ratio greater than 0.6 (CpG Islands in Vertebrate Genomes, http://www.bioinformatics.org/sms2/cpg_islands.html). By intersecting our SNV data with our predicted TATA boxes and CpG islands we were able to analyse putative regulatory region mutations.

All the genes, including micro RNAs, were ranked on the rate of non-synonymous coding SNV (dN/L), rate of non-synonymous to synonymous SNV (dN/dS), rate of exonic SNV, rate of intronic SNV.

#### Pathway analysis

The gene lists in the QTL candidate regions for *Campylobacte*r colonisation resistance were analysed using the IPA programme (www.ingenuity.com) in order to identify canonical pathways and gene networks constructed by the products of the genes located there. IPA constructs many possible upstream regulators, pathways and networks serving as hypotheses for the biological mechanism underlying the data based on a large-scale causal network derived from the Ingenuity Knowledge Base. Then, IPA infers the most suitable pathways and networks based on their statistical significance, thus a threshold above which the pathways are significant is derived.

#### Gene expression analysis

Genes whose expression was studied included *CXCLi1* and *CXCLi2*. Total RNA from a caecal tonsil was isolated from four replicate control birds and eight replicate infected birds per line (6_1_ and N) using the RNeasy Mini Kit (Qiagen West Sussex, United Kingdom) following the manufacturer’s instructions. For each gene, mRNA levels were quantified using TaqMan quantitative RT-PCR. Primers and probes for the 28S rRNA, *CXCLi1* and *CXCLi2* genes were as described previously [78, 79]. TaqMan assays were performed as described by Sutton et al. [80]. Data was expressed as cycle threshold (Ct) value, which was normalised using the formula; Ct + (N′t-C′t)*(S/S′) where N′t is the mean Ct value for 28S RNA among all samples, C′t is the mean value for 28S RNA in the sample and the S and S′ are the slopes of regression of the standard plots for the specific cytokine or chemokine mRNA and the 28S RNA, respectively. Expression data was calculated as fold changes compared with the mean of the control mock-infected samples. Results are expressed as the mean of the fold changes between replicate samples and error bars represent the standard error of this mean. Differences between control and infected samples were examined with an analysis of variance.

## Ethics

All animal experiments were conducted in accordance with the Animals (Scientific Procedures) Act 1986, with the approval of the Ethical Review Committee of The Pirbright Institute (under project licence PPL 30/2462) and the Animal Welfare and Ethical Review Body of The Roslin Institute (under PPL 60/4420).

## Availability of supporting data

The SNP data used in this study is available in NCBI dbSNP at the following web page: http://www.ncbi.nlm.nih.gov/SNP/snp_viewBatch.cgi?sbid=1062063. The same data is also available at the National Avian Research Facility website through the link: http://www.narf.ac.uk/VariationDatabase.

## Additional files

Additional file 1: Table S1 Levels of *C. jejuni* colonisation in the caeca of line 6_1_, line N, back-cross and advanced intercross line birds. The measurements took place one, two, three, four and five days after challenge at 3 weeks of age. Data are presented as mean log_10_ (CFU/g) ± standard deviation of the mean. (XLSX 12 kb) Additional file 2: Figure S1 Manhattan plot and Q-Q plot displaying the GWAS results from the back-cross experiment. Genomic location is plotted against -log_10_(P) in the Manhattan plot (above). Suggestive genome-wide threshold is shown as a horizontal line. Q–Q plot (below) of observed *P*-values against the expected *P*-values for *Campylobacter* gut colonisation (log-transformed number of *C. jejuni* per gram of caecal contents). (PPTX 1422 kb) Additional file 3: Figure S2 Manhattan plot and Q-Q plot displaying the GWAS results from the AIL experiment (binary (0/1) phenotypes). Genomic location is plotted against -log_10_(P). Genome-wide (*P* < 0.05) and suggestive genome-wide thresholds are shown as dashed lines. Q–Q plot (below) of observed *P*-values against the expected *P*-values for *Campylobacter* gut colonisation level. (PPTX 1524 kb) Additional file 4: Table S2 Genes located in the QTL candidate regions for *Campylobacter* colonisation resistance. (XLSX 27 kb) Additional file 5: Figure S3 Linkage disequilibrium (LD) pattern for significant SNPs on chromosome 14. LD between SNPs in the 0.4 Mb on chromosome 14 region. LD blocks are marked with triangles. The significant markers are illustrated with a blue arrow. Strongest LD signals are in red and weakest in white. (PPTX 2065 kb) Additional file 6: Figure S4 Linkage disequilibrium (LD) pattern for significant SNPs on chromosome 4. LD between SNPs in the 0.4 Mb on chromosome 4 region. LD blocks are marked with triangles. The significant marker is illustrated with a blue arrow. Strongest LD signals are in red and weakest in white. (PPTX 1646 kb) Additional file 7: Figure S5 Linkage disequilibrium (LD) block containing the significant SNP identified on chromosome 16. (PPTX 1681 kb) Additional file 8: Figure S6 Summary statistics of the single nucleotide variants identified in the candidate regions for *Campylobacter* colonisation resistance. (PNG 41 kb) Additional file 9: Table S3 List of single nucleotide variants located in the candidate regions for *Campylobacter* colonisation resistance. Variant Effect Predictor annotation results, SIFT predictions and allelic frequencies per each parental line are also presented. (XLSX 1242 kb) Additional file 10: Table S4 Genes located in the QTL candidate regions for *Campylobacter* resistance containing splicing, 5′UTR, both missense and UTR, CpG sites SNVs. (XLSX 14 kb) Additional file 11: Figure S7 Network analysis using IPA software. The two networks (A related to immunological disease and hereditary disorder) and (B related to cell cycle, cell death and survival, connective tissue development and function) illustrate molecular interactions between products of candidate genes selected from QTL regions from the AIL. Arrows with solid lines represent direct interactions, and arrows with broken lines represent indirect interactions. The white colour indicates gene products added to the IPA analysis because of their interaction with the target gene products. (PPTX 1586 kb) Additional file 12: Figure S8 Chemokine gene expression. Quantitation of CXCLi1 and CXCLi2 mRNA expression in caecal tonsils of line 6_1_ and line N *Campylobacter jejuni* infected birds at 5 days post infection. Data are expressed as the fold change in mRNA levels when samples from infected birds were compared to non-infected birds of the same age from each line. Error bars show ± S.E.M. Asterisks indicate significant differences (*P* < 0.05) between line 6_1_ and line N. (PPTX 204 kb) Additional file 13: Table S5 List of selected candidate genes for *Campylobacter* colonisation resistance detected with the back-cross and AIL experiment. Expression of the candidate genes for *Campylobacter* resistance identified in the present study using data from previously published studies of other poultry infections. (XLSX 15 kb) Additional file 14: Table S6 List of SNP markers used in the back-cross analysis. (XLSX 48 kb)

## Abbreviations

AIL: advanced intercross line
*B-BTN2*: Butyrophilin subfamily 2 member A2
*BFIV21*: MHC class I alpha chain II
*BG1*: MHC class IV antigen (B-G)
*BLEC-2*: B-cell CLL/Lymphoma 2
*C. jejuni*: *Campylobacter jejuni*
*C16orf96*: chromosome 16 open reading frame 96
*C4*: complement 4 precursor
*CCDC108*: coiled-coil domain containing 108
*CDH11*: cadherin 11 precursor gene
*CDH13*: T-cadherin
*CDH5*: cadherin 5-type 2
*CDH8*: cadherin 8-type 2
CFU: colony-forming units
dN/dS: rate of non-synonymous to synonymous SNV
dN/L: number of non-synonymous SNV divided by the length of the coding region of the gene
*EPHA5*: ephrin receptor A5
*GLB1L*: galactosidase, beta 1-like
GWAS: genome-wide association study
IBDV: infectious bursal disease virus
IBV: infectious bronchitis virus
*IFIH1*: interferon-induced helicase C domain-containing protein 1
*IL4I1*: interleukin 4 Induced 1
LD: linkage disequilibrium
*LY75*: lymphocyte antigen 75 precursor
MAF: minor allele frequency
MDV: Marek’s disease virus
MHC: major histocompatibility complex
MSA: multidimensional scaling analysis
*NAT15*: N-acetyltransferase 15
*NRAMP1 (SLC11A1)*: natural resistance-associated macrophage protein 1
*OBSL1*: Obscurin-like 1
*PTPRN*: protein tyrosine phosphatase, receptor type, N
QTL: quantitative trait loci
*RACGAP1*: Rac GTPase-activating protein 1
*SLC38A11*: solute carrier family 38, member 11
SNP: single nucleotide polymorphism
SNV: single nucleotide variant
*SPEG*: SPEG complex locus
SPF: specified pathogen-free
*TAP2*: antigen peptide transporter 2
*TRAP*: Tumour necrosis factor receptor-associated protein 1
*TRIM 27*: Tripartite motif–containing 27
*TTC21B*: Tetratricopeptide repeat domain 21B
UTR: untranslated region
*ZNF142*: Zinc finger protein 142
