# Supplementary figures and images for: The genomic architecture of resistance to Campylobacter jejuni intestinal colonisation in chickens
Source: BMC Genomics. 2016 Apr 18;17:293. doi: 10.1186/s12864-016-2612-7 (PMC4835825; doi:10.1186/s12864-016-2612-7)

## Slide 1
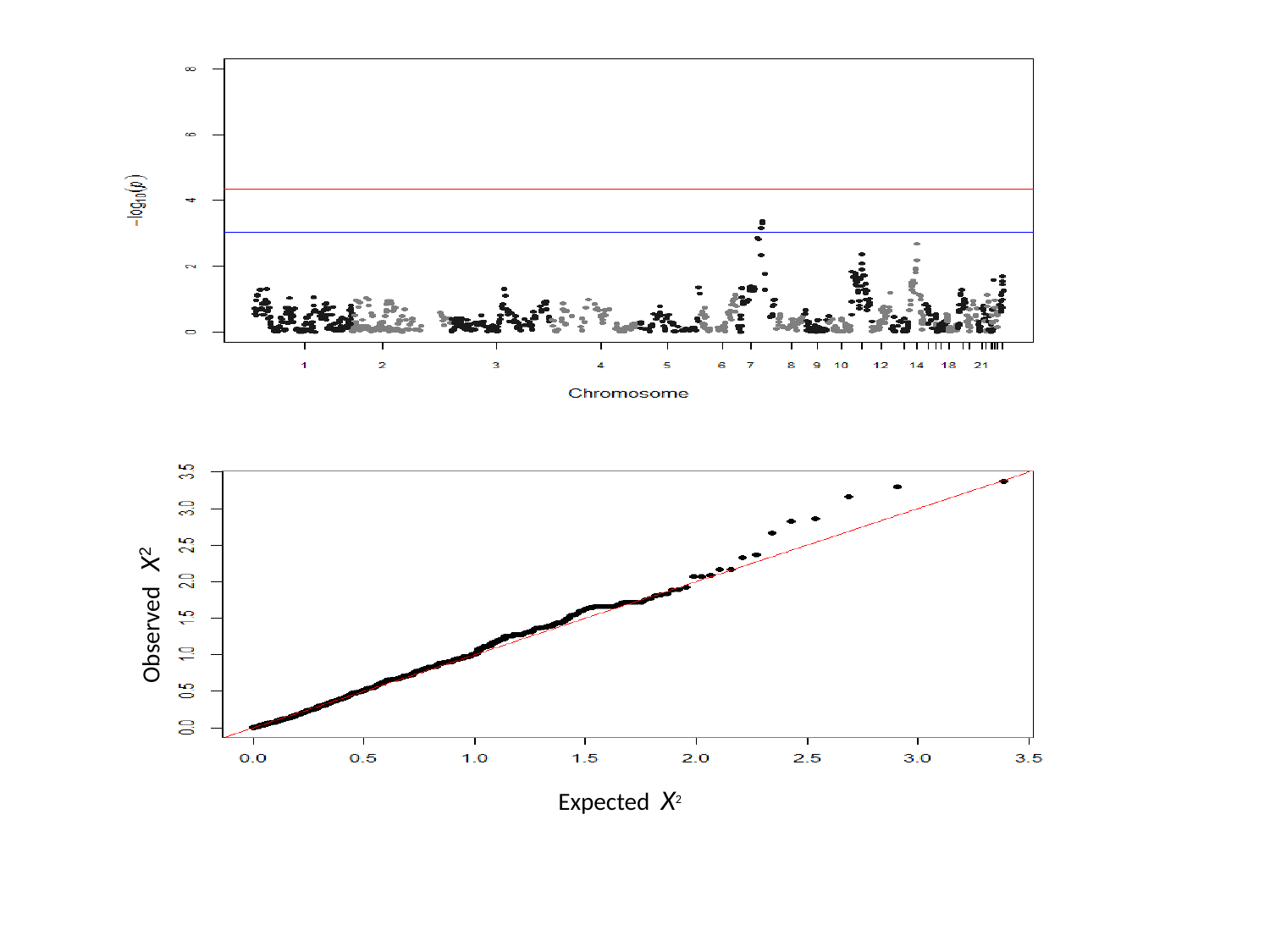

Observed X2
Expected X2

Supplement: Additional file 2: Figure S1 — Manhattan plot and Q-Q plot displaying the GWAS results from the back-cross experiment. Genomic location is plotted against -log10(P) in the Manhattan plot (above). Suggestive genome-wide threshold is shown as a horizontal line. Q–Q plot (below) of observed P-values against the expected P-values for Campylobacter gut colonisation (log-transformed number of C. jejuni per gram of caecal contents). (PPTX 1422 kb) [file 12864_2016_2612_MOESM2_ESM.pptx]

## Slide 1
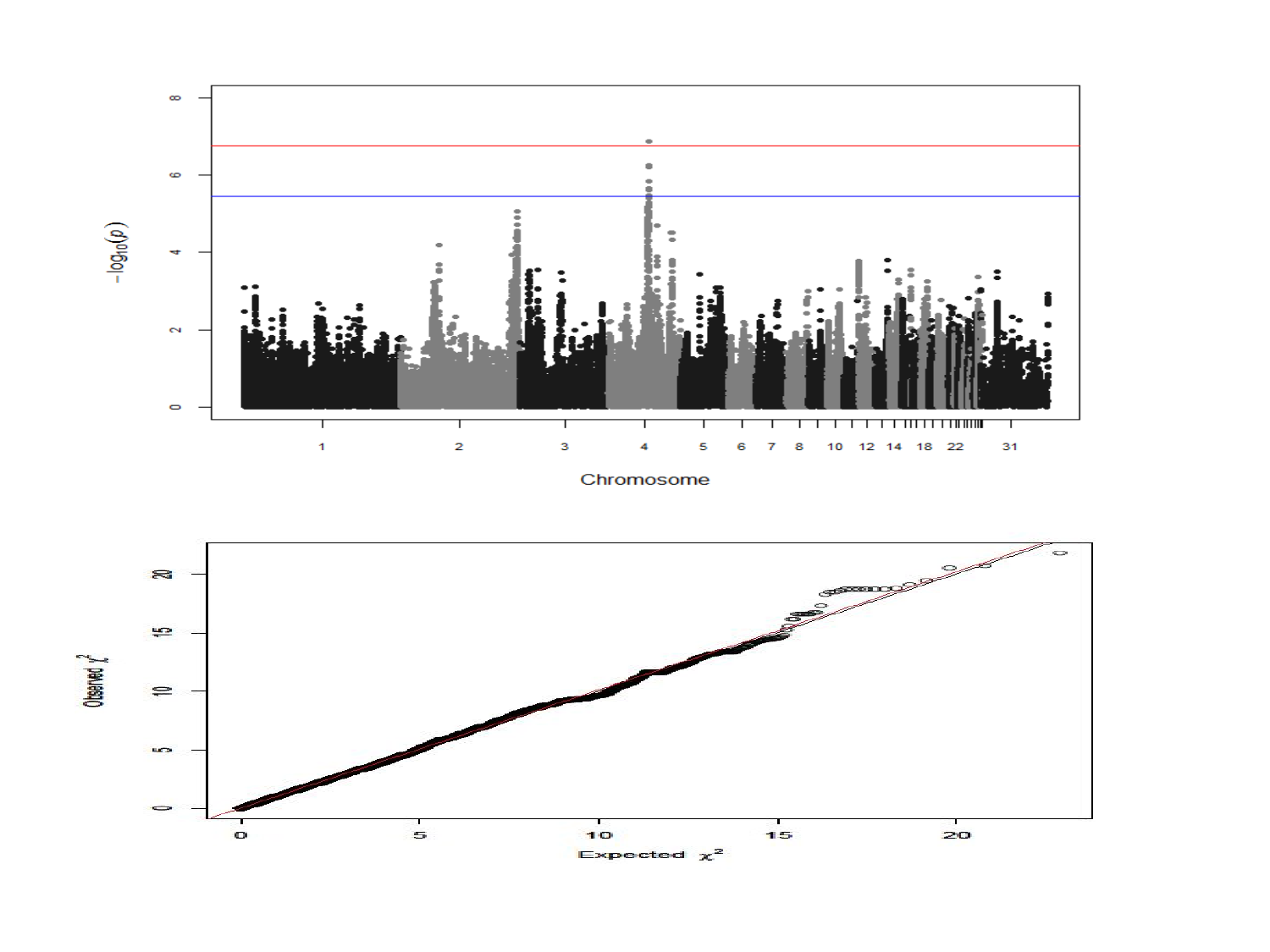

Supplement: Additional file 3: Figure S2 — Manhattan plot and Q-Q plot displaying the GWAS results from the AIL experiment (binary (0/1) phenotypes). Genomic location is plotted against -log10(P). Genome-wide (P < 0.05) and suggestive genome-wide thresholds are shown as dashed lines. Q–Q plot (below) of observed P-values against the expected P-values for Campylobacter gut colonisation level. (PPTX 1524 kb) [file 12864_2016_2612_MOESM3_ESM.pptx]

## Slide 1
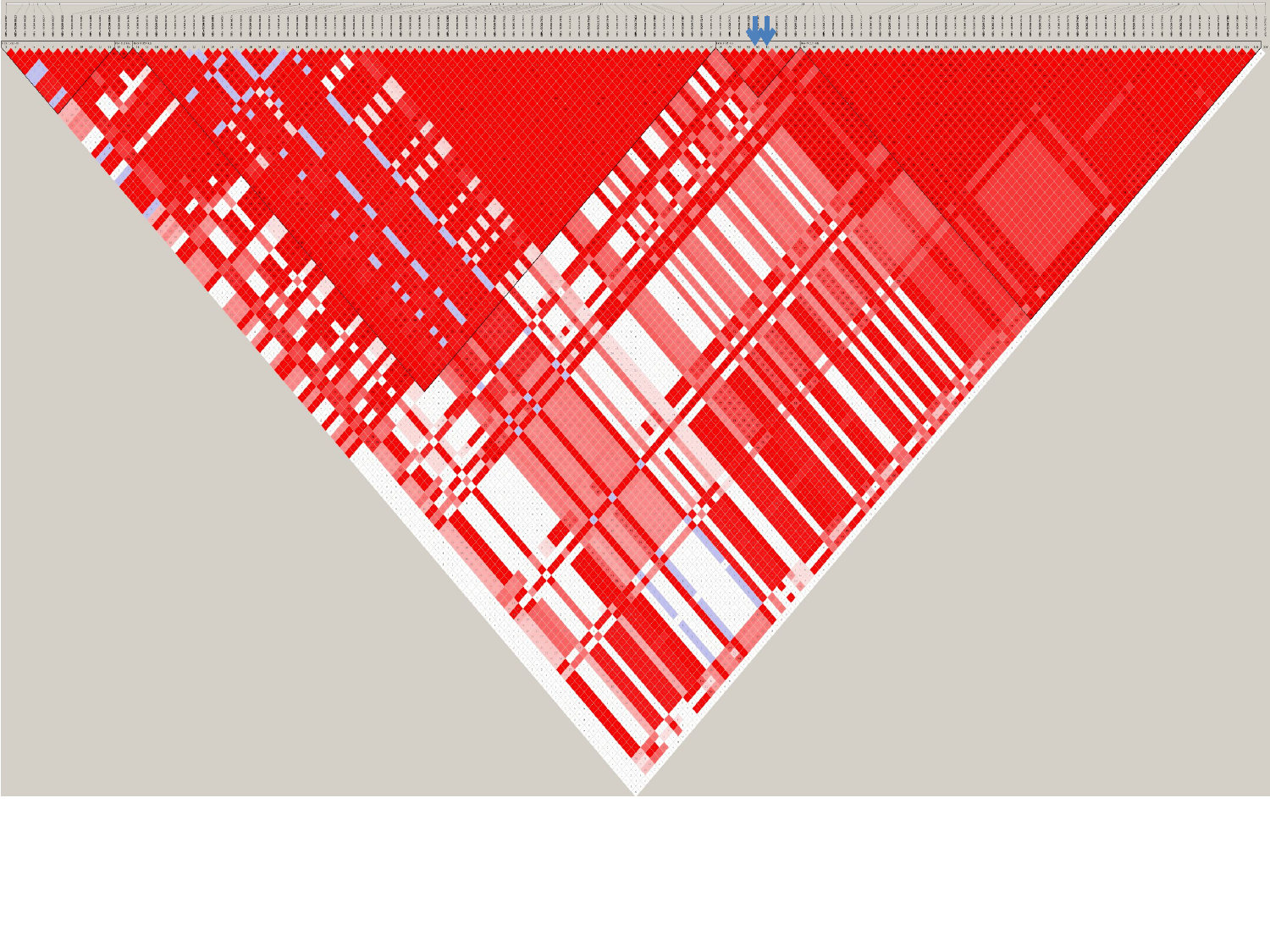

Supplement: Additional file 5: Figure S3 — Linkage disequilibrium (LD) pattern for significant SNPs on chromosome 14. LD between SNPs in the 0.4 Mb on chromosome 14 region. LD blocks are marked with triangles. The significant markers are illustrated with a blue arrow. Strongest LD signals are in red and weakest in white. (PPTX 2065 kb) [file 12864_2016_2612_MOESM5_ESM.pptx]

## Slide 1
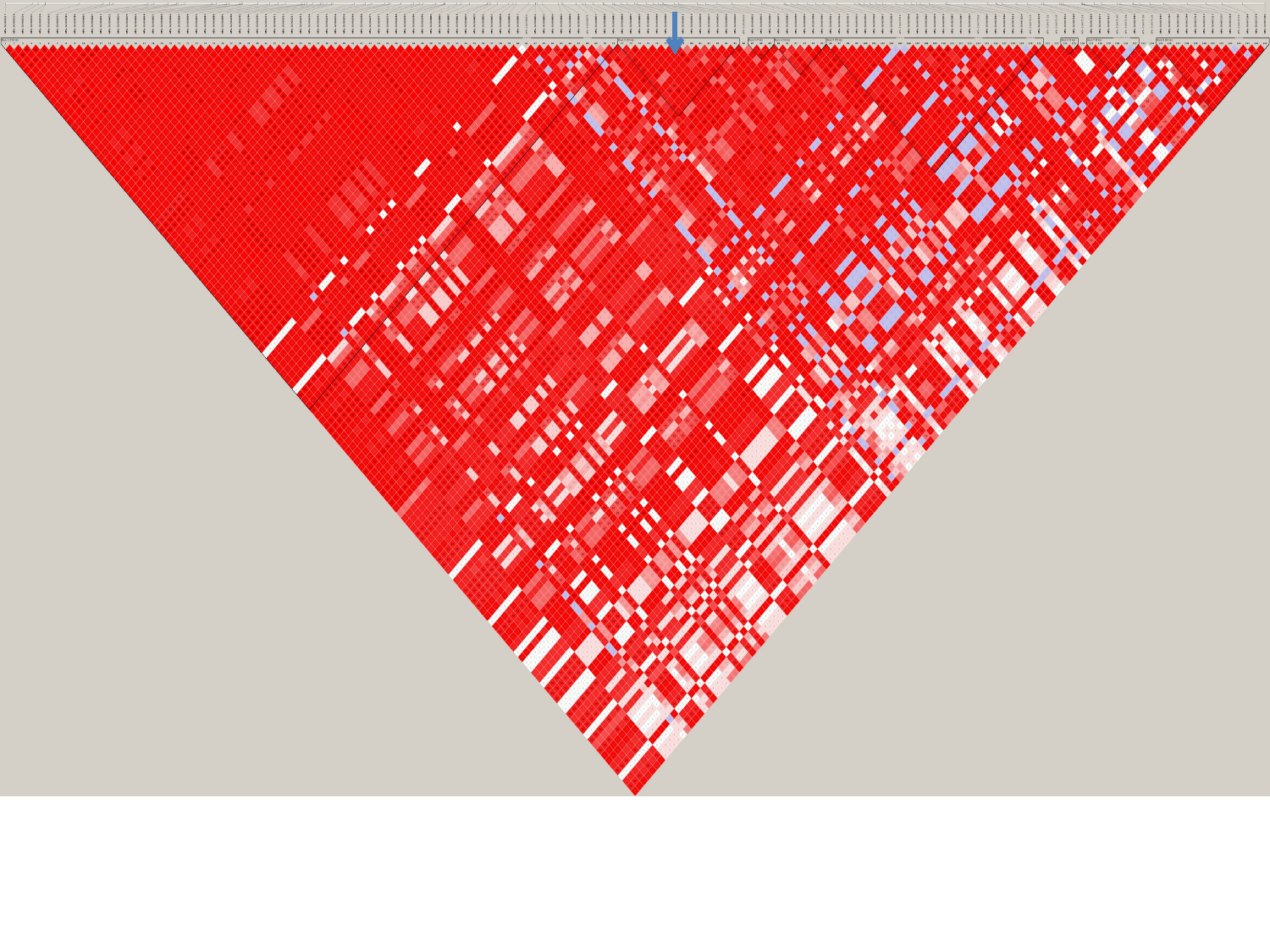

Supplement: Additional file 6: Figure S4 — Linkage disequilibrium (LD) pattern for significant SNPs on chromosome 4. LD between SNPs in the 0.4 Mb on chromosome 4 region. LD blocks are marked with triangles. The significant marker is illustrated with a blue arrow. Strongest LD signals are in red and weakest in white. (PPTX 1646 kb) [file 12864_2016_2612_MOESM6_ESM.pptx]

## Slide 1
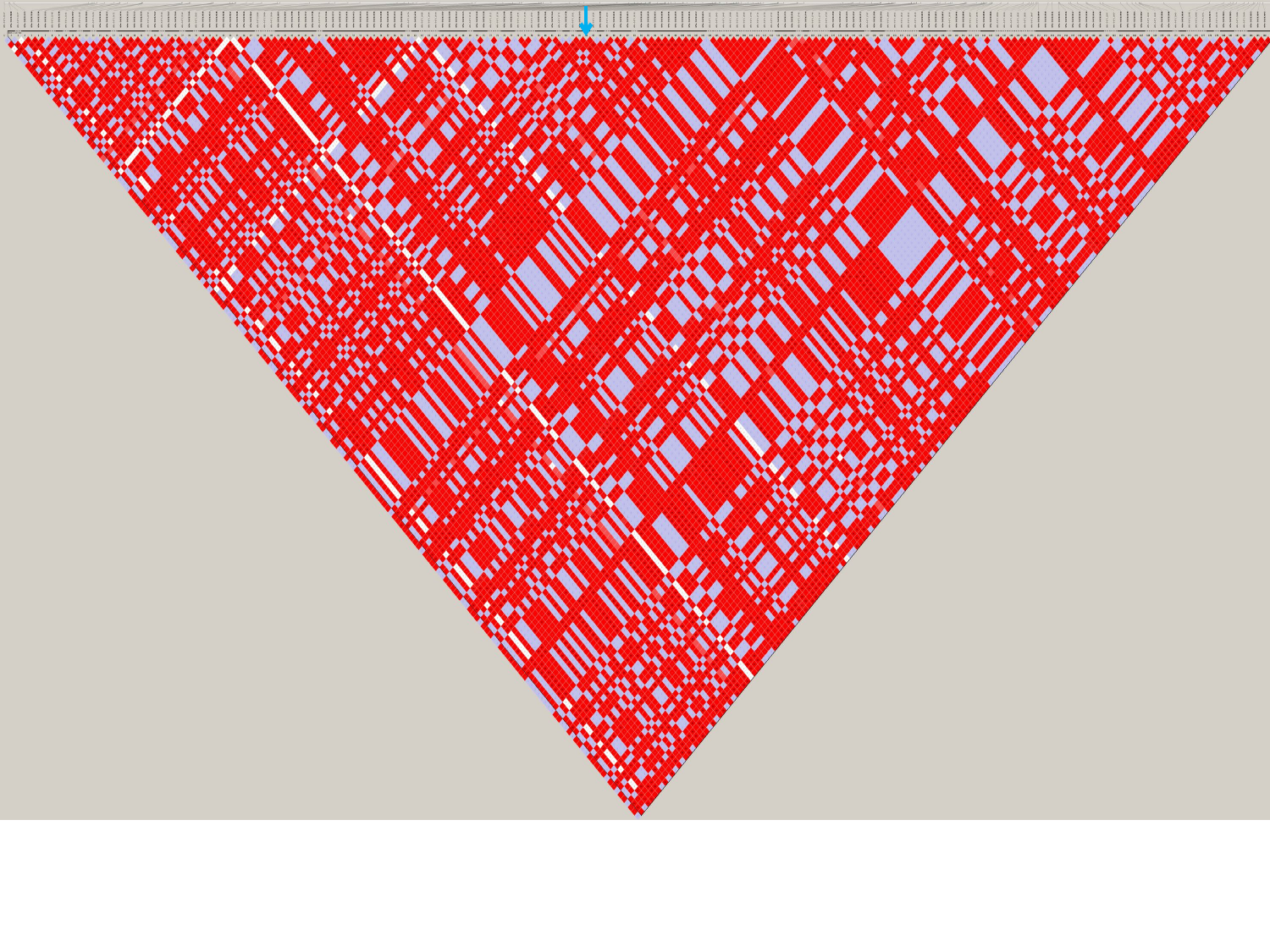

Supplement: Additional file 7: Figure S5 — Linkage disequilibrium (LD) block containing the significant SNP identified on chromosome 16. (PPTX 1681 kb) [file 12864_2016_2612_MOESM7_ESM.pptx]

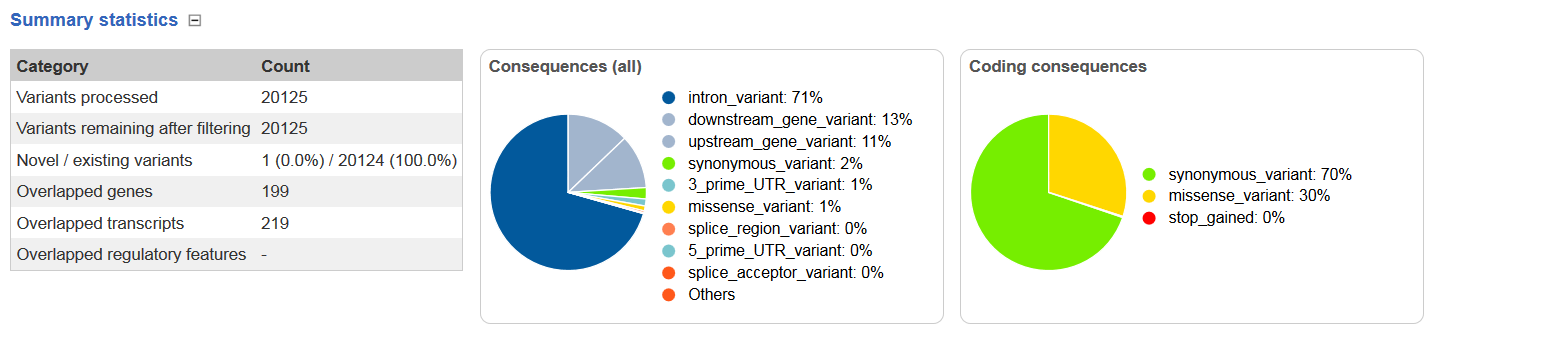

Supplement: Additional file 8: Figure S6 — Summary statistics of the single nucleotide variants identified in the candidate regions for Campylobacter colonisation resistance. (PNG 41 kb) [file 12864_2016_2612_MOESM8_ESM.png]

## Slide 1
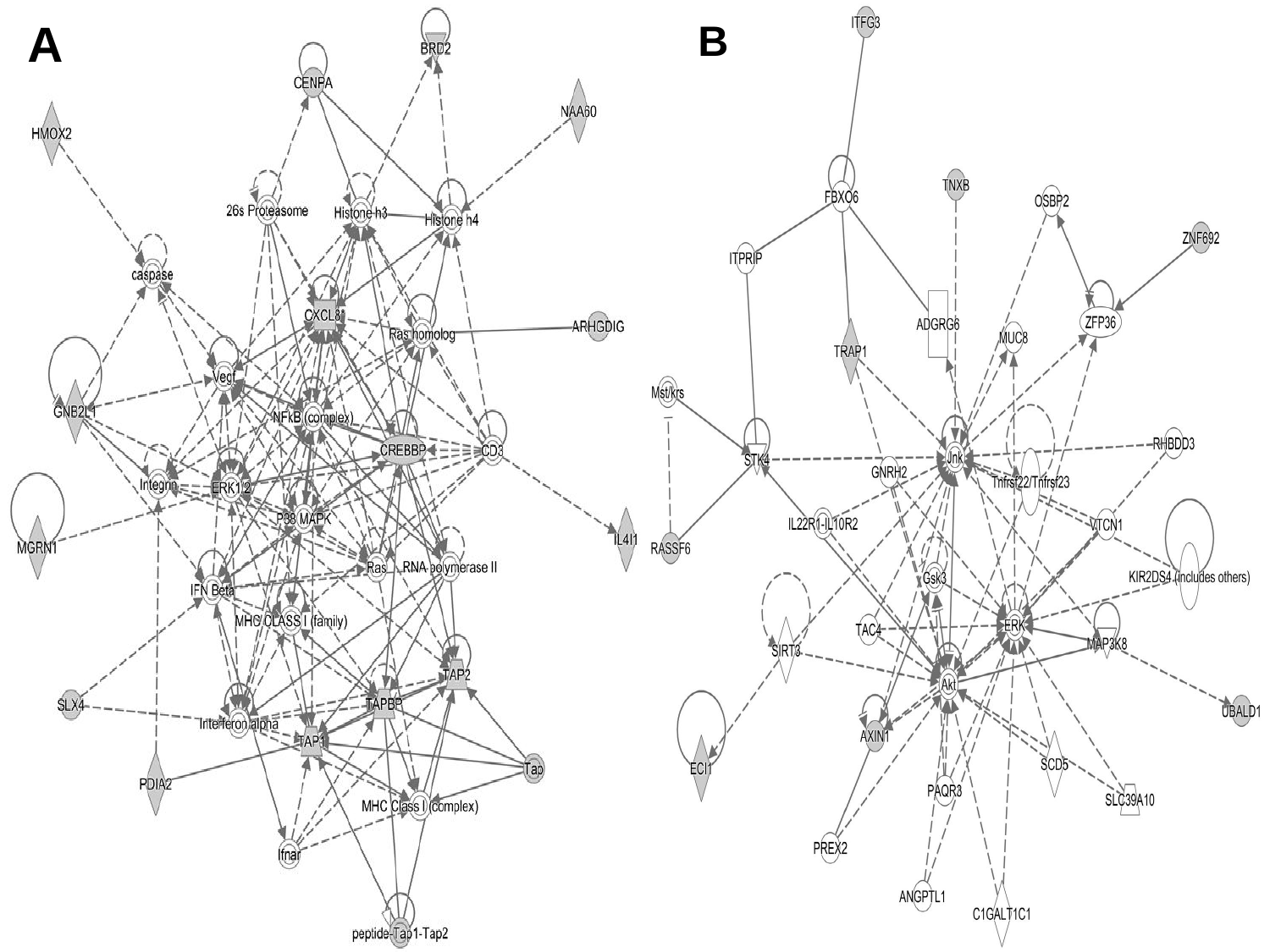

A
B
#

Supplement: Additional file 11: Figure S7 — Network analysis using IPA software. The two networks (A related to immunological disease and hereditary disorder) and (B related to cell cycle, cell death and survival, connective tissue development and function) illustrate molecular interactions between products of candidate genes selected from QTL regions from the AIL. Arrows with solid lines represent direct interactions, and arrows with broken lines represent indirect interactions. The white colour indicates gene products added to the IPA analysis because of their interaction with the target gene products. (PPTX 1586 kb) [file 12864_2016_2612_MOESM11_ESM.pptx]

## Slide 1
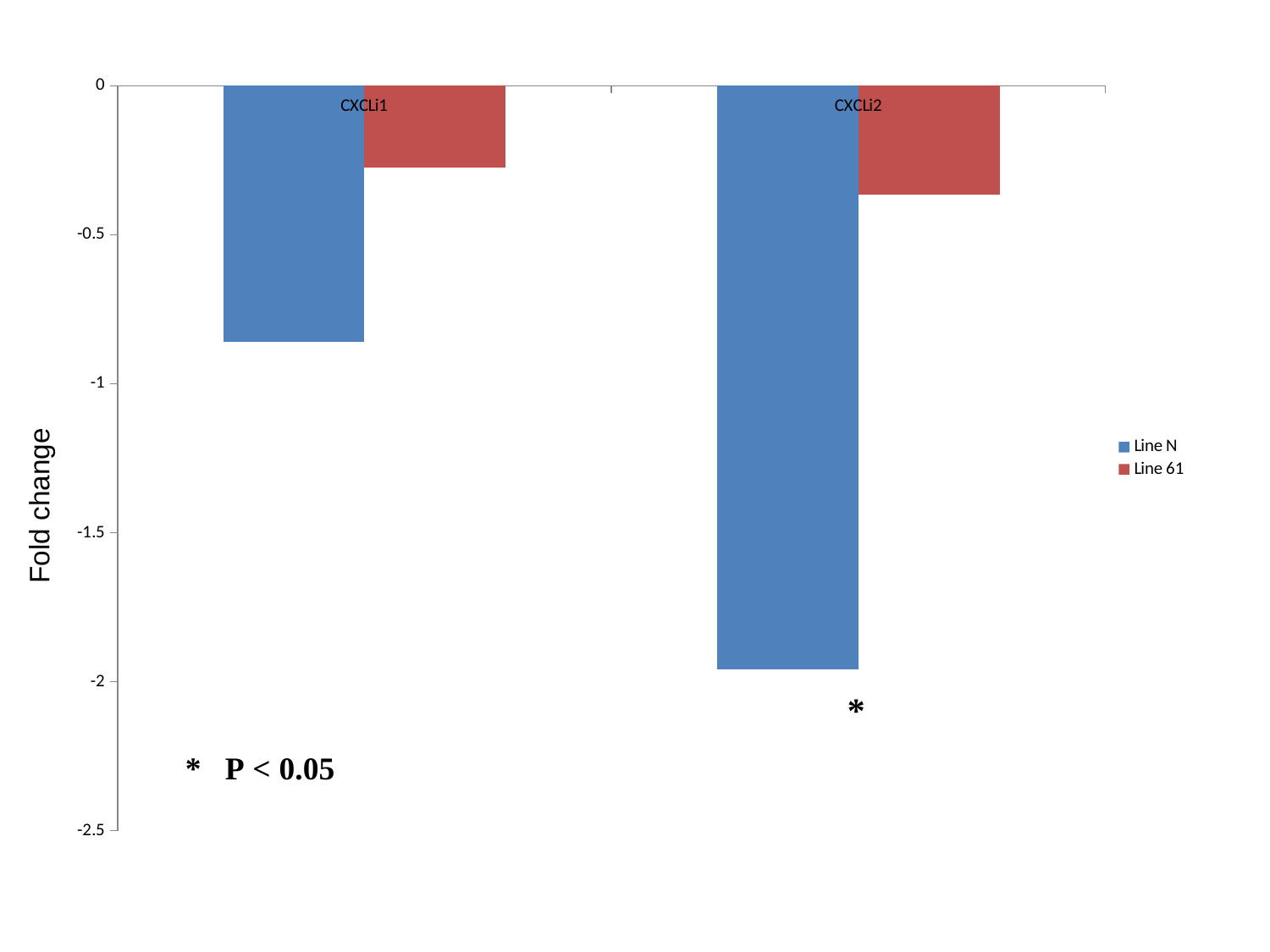

### Chart
| Category | Line N | Line 61 |
|---|---|---|
| CXCLi1 | -0.85832193 | -0.274502289 |
| CXCLi2 | -1.959964432 | -0.364344033 |Fold change
*

Supplement: Additional file 12: Figure S8 — Chemokine gene expression. Quantitation of CXCLi1 and CXCLi2 mRNA expression in caecal tonsils of line 61 and line N Campylobacter jejuni infected birds at 5 days post infection. Data are expressed as the fold change in mRNA levels when samples from infected birds were compared to non-infected birds of the same age from each line. Error bars show ± S.E.M. Asterisks indicate significant differences (P < 0.05) between line 61 and line N. (PPTX 204 kb) [file 12864_2016_2612_MOESM12_ESM.pptx]
